# Live Birth Following Dibutyryl‐cAMP‐Enhanced Biphasic in Vitro Maturation of Ovarian Tissue Oocytes From a Patient With Ovarian Fibromatosis: A First Report

**DOI:** 10.1002/rmb2.70024

**Published:** 2026-03-11

**Authors:** Shotaro Higuchi, Tsutomu Miyamoto, Miho Mochizuki, Tamae Fukushima, Koichi Ida, Hisanori Kobara, Ayumi Ohya, Yasunari Fujinaga, Tanri Shiozawa

**Affiliations:** ^1^ Department of Obstetrics and Gynecology Shinshu University School of Medicine Nagano Japan; ^2^ Center for Reproductive Medicine Shinshu University Hospital Nagano Japan; ^3^ Department of Radiology Shinshu University School of Medicine Nagano Japan

**Keywords:** cyclic AMP, fertility preservation, in vitro maturation techniques, ovarian fibromatosis, polycystic ovary syndrome

## Abstract

**Case:**

A 23‐year‐old nulligravida with bilateral ovarian fibromatosis, amenorrhea, and severe ovulatory dysfunction was diagnosed via laparoscopic biopsy. As pregnancy was desired but transvaginal oocyte retrieval was not feasible due to dense fibrosis, partial ovarian resection was performed, and oocytes were obtained using ovarian tissue oocyte in vitro maturation (OTO‐IVM).

**Outcome:**

In the first OTO‐IVM cycle with human chorionic gonadotropin (hCG) priming, 11 mature and 63 immature oocytes were retrieved. Mature oocytes underwent intracytoplasmic sperm injection (ICSI), whereas immature oocytes were cultured in vitro for maturation before ICSI. A total of 13 oocytes were fertilized, five embryos were cryopreserved, and one embryo transfer resulted in a biochemical pregnancy. At age 28, a second OTO‐IVM using dibutyryl‐cyclic AMP (dbcAMP)‐enhanced biphasic IVM produced seven germinal vesicle oocytes; three matured to metaphase II, two fertilized, and one cleavage‐stage embryo led to a live birth at 39 weeks of gestation.

**Conclusion:**

This is the first documented live birth following dbcAMP‐enhanced biphasic IVM of ovarian tissue‐derived oocytes. These findings suggest that dbcAMP‐enhanced biphasic IVM may be a feasible option when granulosa cell support is limited, offering a potential addition to fertility preservation strategies.

## 1 Introduction

Ovarian fibromatosis (OF) is a rare, benign ovarian disorder characterized by stromal proliferation and dense fibrous tissue encasing morphologically normal structures. Since its first description in 1984 [1], only approximately 30 cases have been reported [2]. OF typically affects women of reproductive age and may mimic polycystic ovary syndrome (PCOS); however, published reports rarely address reproductive management [3, 4]. The firm, fibrotic nature of the ovaries often renders them resistant to puncture, making conventional transvaginal oocyte retrieval technically infeasible [5]. In these cases, ovarian tissue oocyte in vitro maturation (OTO‐IVM), in which immature oocytes are collected from surgically resected ovarian tissue and matured in vitro, represents an alternative fertility‐preserving strategy [6]. However, clinical outcomes have been limited, largely due to the low number of retrievable oocytes and suboptimal maturation rates.

To overcome the limitations of conventional in vitro maturation (IVM), a biphasic strategy known as capacitation IVM (CAPA‐IVM) was developed [7]. This approach incorporates a pre‐IVM designed to preserve intra‐oocyte cyclic AMP (cAMP), delay meiotic resumption, and better synchronize nuclear and cytoplasmic maturation. In human CAPA‐IVM, C‐type natriuretic peptide (CNP) is typically used to enhance cyclic guanosine monophosphate (cGMP) production in granulosa cells, thereby preventing cAMP degradation and maintaining meiotic arrest (Supplemental Figure S1). However, this mechanism depends heavily on intact granulosa‐oocyte gap junctions, which may be markedly reduced in fibrotic ovaries or in denuded/minimally cumulus‐enclosed oocytes obtained by mechanical retrieval. Dibutyryl‐cAMP (dbcAMP), a cell‐permeable cAMP analog, can sustain meiotic arrest independently of granulosa cells, providing an alternative strategy under such conditions. Although dbcAMP and related cAMP modulators have been evaluated in animal models and in preclinical studies using human oocytes [8, 9], their application in clinical ART cycles leading to embryo transfer or live birth remains very limited.

Here, we report the first documented live birth following dbcAMP‐enhanced biphasic IVM of ovarian tissue‐derived oocytes in patients with OF. This case highlights the potential of dbcAMP‐based biphasic IVM as a granulosa cell‐independent alternative to CNP‐based systems, particularly in patients with atypical ovarian pathologies or limited follicular support.

## 2 Case Report

### 2.1 Patient Background and Diagnosis

**FIGURE 1:**
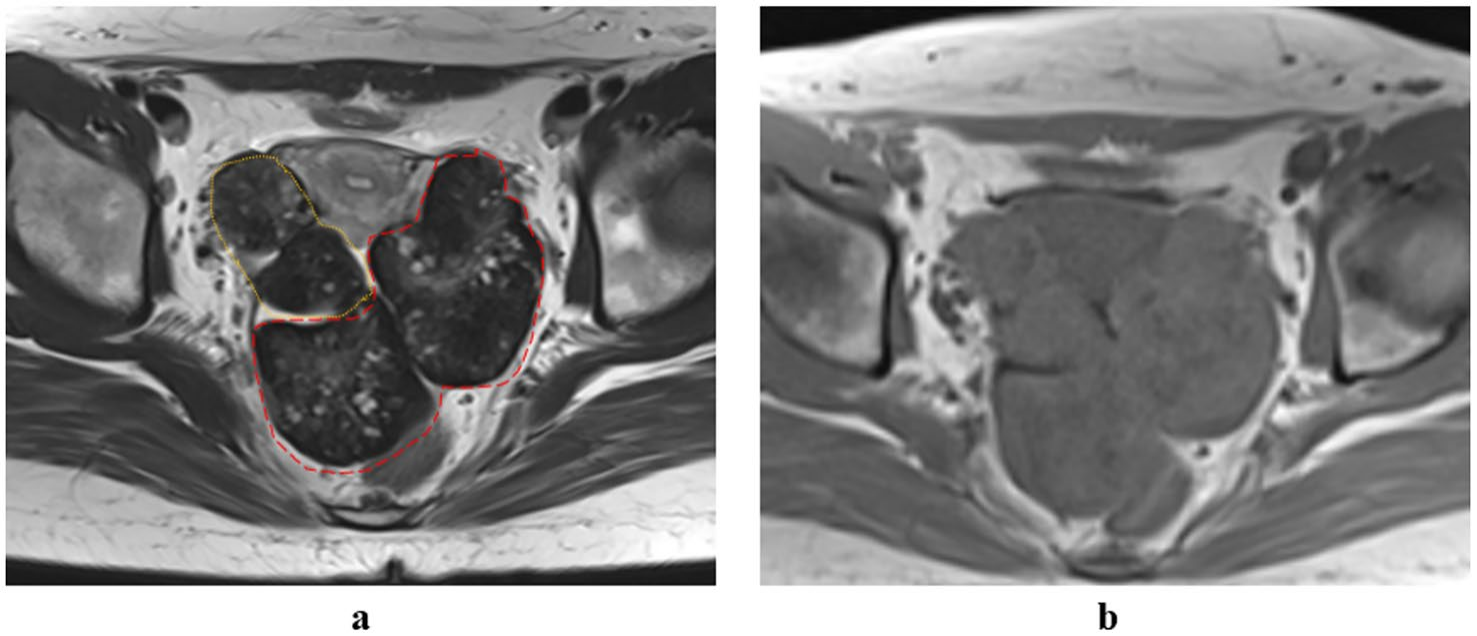
Magnetic resonance imaging findings of the present case. (a) T2‐weighted image, (b) T1‐weighted image. Solid masses were observed in both ovaries. The right (thin‐dotted line) and left (wide‐dotted line) ovaries contain small follicles and are surrounded by low signals on T2‐weighted imaging, suggesting stromal proliferation.

The clinical course of the patient, including key surgical interventions, fertility treatments, and outcomes, is summarized in Figure SS2. A 23‐year‐old nulligravida with irregular menses since menarche presented with a complaint of pelvic mass. Magnetic resonance imaging (MRI) revealed large, lobulated ovarian masses with a hypointense peripheral rim (“black garland sign”) corresponding to fibrotic tissue (Figure 1), suggestive of OF [4]. Laparoscopic biopsy revealed smooth, firm, bilateral ovarian masses, and histopathological examination confirmed the diagnosis of OF. Hormonal evaluation after withdrawal bleeding revealed a PCOS‐like pattern (E2, 56.1 pg/mL; LH, 14.4 mIU/mL; FSH, 3.7 mIU/mL; total testosterone, 0.63 ng/mL) without clinical hyperandrogenism. Following the biopsy, spontaneous ovulation occurred, and Holmström therapy was initiated. At 24 years of age, the patient married and expressed a desire to become pregnant.

### 2.2 Postoperative Course and Fertility Treatment Strategy

One year after the biopsy, MRI demonstrated stable ovarian size, and serum AMH levels remained elevated (27.40 ng/mL). Her husband's semen analysis results were normal. Gonadotropin ovulation induction was considered unsuitable because ultrasound visualization was hindered by dense fibrotic stroma, and elevated AMH levels indicated a high risk of ovarian hyperstimulation syndrome (OHSS). Transvaginal oocyte retrieval was technically unfeasible because of ovarian firmness. Therefore, OTO‐IVM has been proposed as an alternative strategy. This approach had been previously reviewed and approved by the Institutional Review Board of our institution based on accumulated clinical experience at our department, and written informed consent was obtained from the patient prior to treatment (Approval No. B0644; July 12, 2021).

### 2.3 hCG‐Primed IVM From Ovarian Tissue Oocyte

**FIGURE 2:**
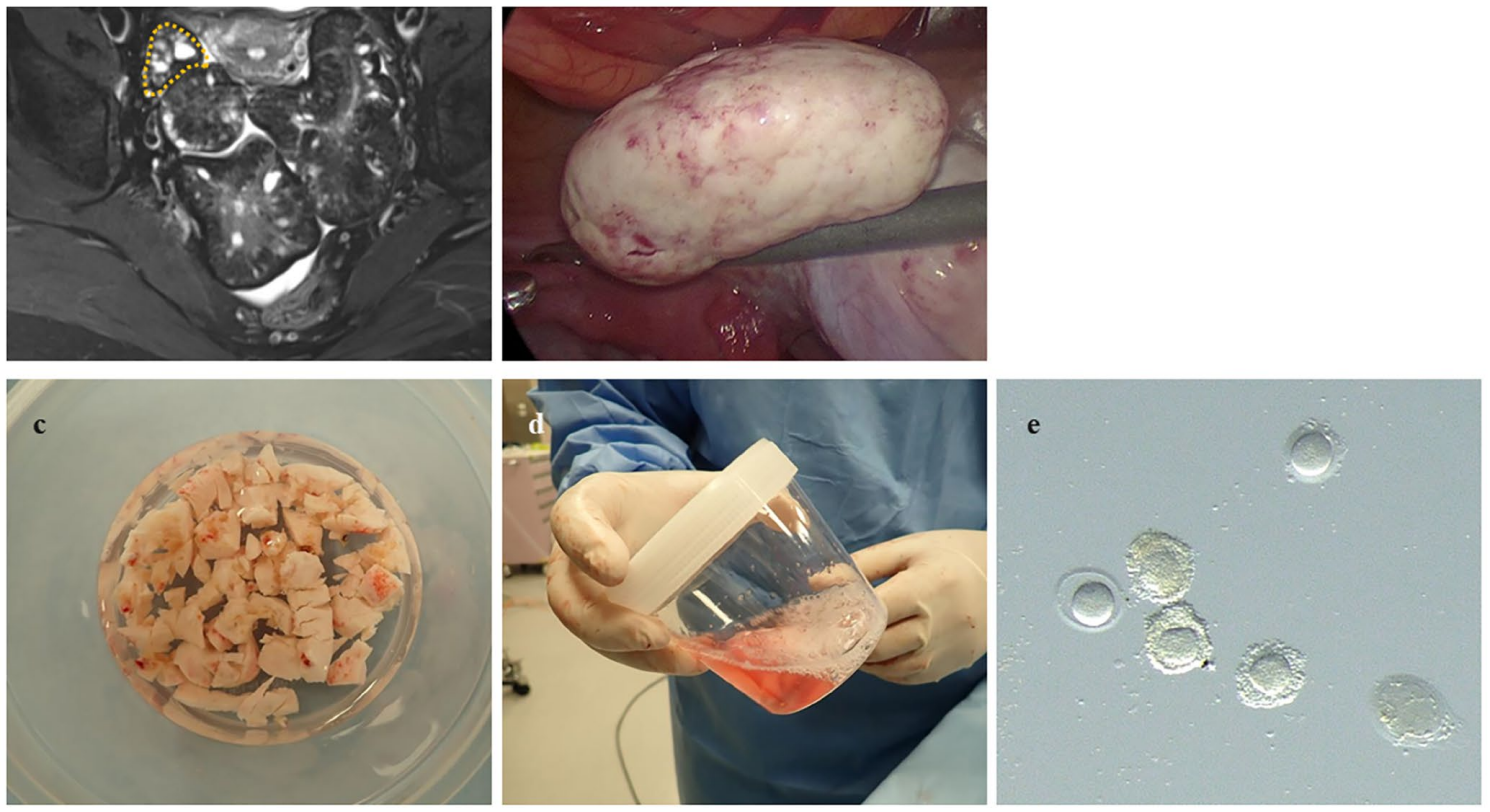
Oocyte retrieval from resected ovarian tissue. (a) Preoperative MRI (T2‐weighted image) identifying the planned resection area within the thumb‐sized (dotted line) portion of the right ovary. (b) Laparoscopic findings of the resected ovarian portion. (c) Diced ovarian tissue after resection, fragmented into 5‐mm cubic pieces. (d) Tissue pieces agitated in culture medium to facilitate oocyte retrieval. (e) Retrieved oocytes from resected ovarian tissue. Oocytes showed minimal cumulus cell attachment, characteristic of ovarian fibromatosis.

**FIGURE 3:**
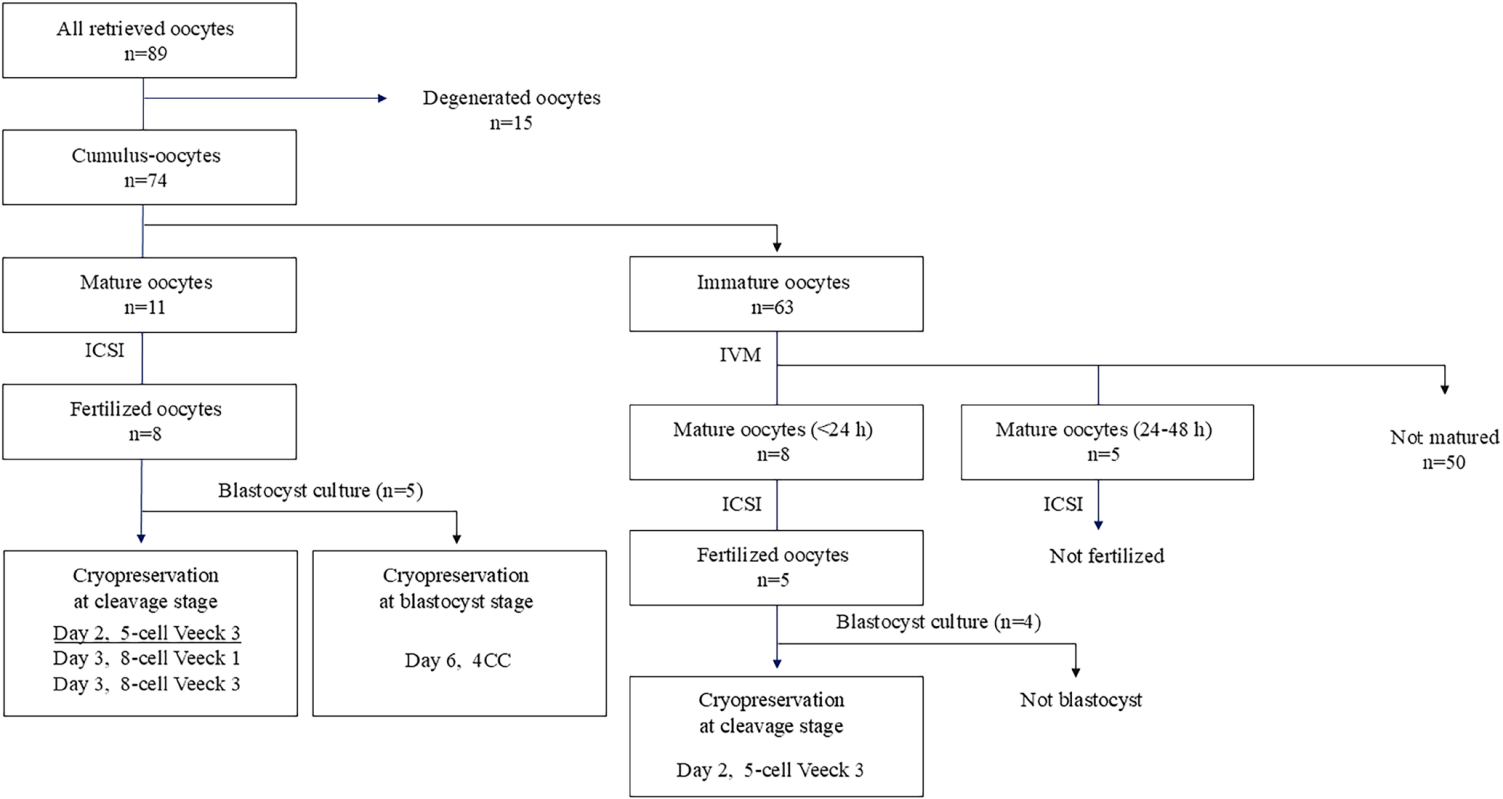
Results of first OTO‐IVM, ICSI, and cryopreservation of retrieved oocytes. The flowchart summarizes the outcomes of oocyte maturation, fertilization, and cryopreservation. A total of 89 oocytes were retrieved from the diced ovarian tissue, of which 74 were viable. Of these, 11 oocytes were mature at retrieval and underwent immediate ICSI, resulting in eight fertilized embryos. The remaining 63 immature oocytes were subjected to IVM, leading to the maturation of 13 oocytes (eight matured within 24 h and five within 24–48 h). After ICSI, five fertilized embryos were obtained. Among the fertilized embryos, four cleavage‐stage embryos (including a 5‐cell Veeck Grade 3 embryo) and one blastocyst were successfully cryopreserved. All embryos were transferred; however, only the underlined embryo was implanted, which resulted in a biochemical pregnancy.

Ovarian stimulation was not performed. Instead, 10,000 IU of hCG was administered intramuscularly 35 h before laparoscopic ovarian tissue resection to promote in vivo oocyte maturation. The resected portion of the right ovary (38 × 26 × 21 mm on preoperative MRI) was processed immediately after excision (Figure 2A,B). Individual follicles could not be identified or aspirated because of the dense fibrotic texture of the ovaries. The tissue was then minced into ~5 mm fragments and agitated in the culture medium (Figure 2C,D), yielding 89 oocytes, of which 74 were viable, including 11 mature oocytes. All the retrieved oocytes exhibited minimal cumulus cell attachment, consistent with OF pathology (Figure 2E). Mature oocytes underwent immediate ICSI, resulting in eight fertilized oocytes. The remaining 63 immature oocytes were cultured in IVM medium (Kitazato Inc., Shizuoka, Japan) under 5% O_2_ and 6% CO_2_ at 37.0°C. After 24–48 h, 13 additional oocytes reached the metaphase II (MII) stage (eight within 24 h; five within 24–48 h) and were fertilized via ICSI using cryopreserved sperm, resulting in five fertilized oocytes. Cleavage‐stage embryos with appropriate morphology and timing were cryopreserved, and the remaining embryos were cultured to the blastocyst stage (Figure 3). Four cleavage‐stage embryos and one blastocyst were vitrified for this study. Of the 13 embryos, 11 (84.6%) exhibited direct cleavage during their first division. One embryo without direct cleavage was cryopreserved on day two as a 5‐cell Veeck Grade 3 embryo. The postoperative course was uneventful. Histopathological examination confirmed OF without malignant features. After 3 months, a single embryo transfer was performed during the hormone‐replacement cycle. The transferred embryo was a 5‐cell Veeck Grade 3 embryo derived from mature oocytes retrieved on the day of surgery. Serum hCG initially rose to 281 mIU/mL but subsequently declined, and the pregnancy was diagnosed as a biochemical pregnancy. The remaining embryos did not result in pregnancy, and the patient opted to continue with fertility treatment.

### 2.4 dbcAMP‐Enhanced Biphasic‐IVM From Ovarian Tissue Oocyte

The patient underwent a second OTO‐IVM cycle at the age of 28 years. In the first cycle, most retrieved oocytes showed minimal cumulus cell attachment, and the resulting oocytes exhibited poor developmental competence. Therefore, we modified the culture system from a single‐step IVM protocol to a biphasic IVM approach to improve oocyte and embryo quality. As illustrated in Figure SS1, a CNP‐based pre‐IVM system maintains intra‐oocyte cAMP levels through granulosa‐oocyte gap junctions; however, this mechanism is unlikely to be effective for denuded or minimally supported oocytes typically obtained from ovarian fibromatosis tissue.

The use of dbcAMP for pre‐IVM was specifically reviewed and approved by the Institutional Review Board of Shinshu University Hospital (Approval No. B0981; June 10, 2024). The committee acknowledged that dbcAMP has been widely used in livestock IVM systems and in preclinical experiments with human oocytes, but has not previously been incorporated into clinical ART cycles, leading to embryo transfer or live birth; therefore, its fetal safety profile remains uncertain. Although no toxic effects have been reported in animal studies, other approaches are unlikely to meaningfully improve oocyte quality in this patient, given the extremely limited granulosa cell support. After a detailed discussion, the IRB concluded that dbcAMP could be used only after full disclosure of its experimental nature and potential risks, followed by renewed written informed consent.

The patient and her husband received comprehensive counseling regarding.

(1) the lack of established human safety data,

(2) the theoretical risks to future offspring,

(3) the rationale for selecting dbcAMP in the context of insufficient cumulus support, and,

(4) the available alternative options.

Written informed consent, specifically for the use of dbcAMP‐enhanced biphasic IVM, was obtained before the cycle. We initially divided the retrieved oocytes into two groups for internal comparison: single‐step IVM and dbcAMP‐enhanced biphasic IVM.

**FIGURE 4:**
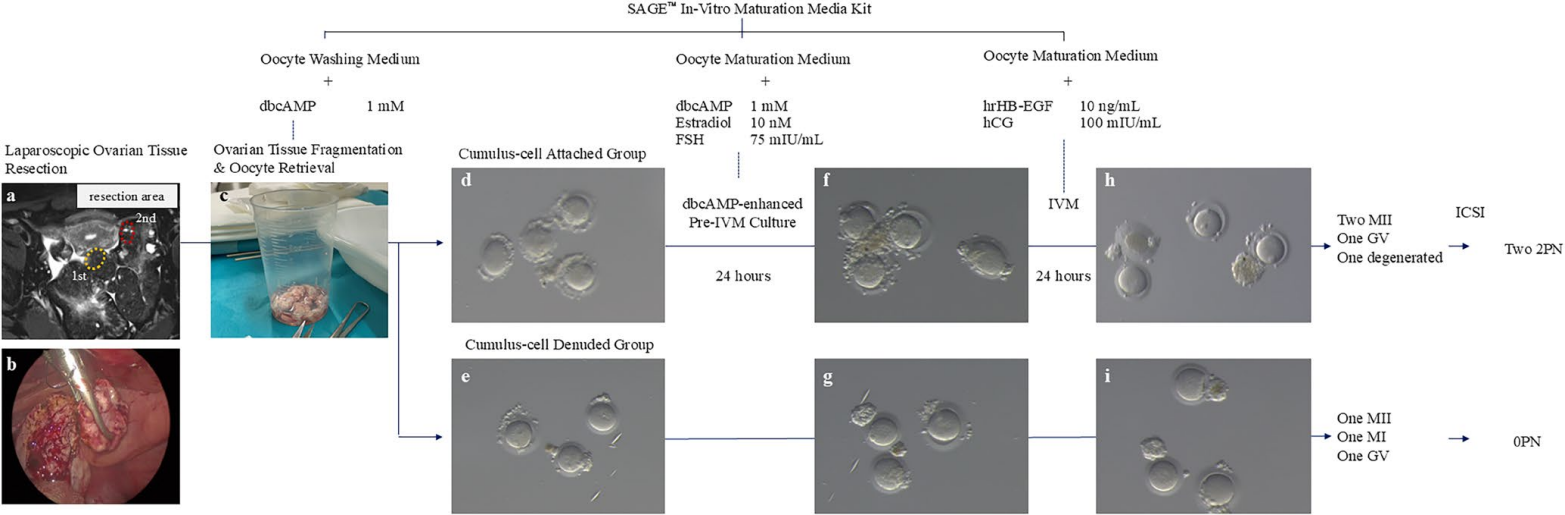
Workflow of dbcAMP‐enhanced biphasic‐IVM using oocytes retrieved from resected ovarian tissue. (a) T2‐weighted MR image showing the resection areas of the left ovary. The first and second resection sites are indicated by yellow‐ and red‐dotted circles, respectively. (b) Intraoperative image of laparoscopic ovarian tissue resection. (c) Retrieved ovarian tissue after fragmentation for oocyte isolation. (d, e) Representative images of oocytes immediately after retrieval, classified into two groups: Cumulus‐cell Attached Group (CC+) (d) and Cumulus‐cell denuded group (CC−) (e). (f, g) Oocytes after dbcAMP‐enhanced Pre‐IVM culture (24 h). (h, i) Oocytes following IVM culture (24 h) and their final maturation outcomes. In the CC+ group, two oocytes matured to MII, one remained at the GV stage, and one degenerated. Two oocytes were fertilized using ICSI, and one embryo with normal cleavage was cryopreserved. In the CC− group, one oocyte matured to MII, one arrested at MI, and one remained at the GV stage. CC−, Cumulus‐cell Denuded Group; CC+, Cumulus‐cell Attached Group; dbcAMP, Dibutyryl cyclic AMP; GV, Germinal Vesicle; IVM, In Vitro Maturation; MI, Metaphase I; MII, Metaphase II.

At the time of the second cycle, the serum AMH was 16.63 ng/mL, and MRI showed no progression in the ovarian mass size. Given the presence of multiple antral follicles, repeated oocyte retrieval from the right ovary was considered for this patient. However, to avoid the potential risk of fertility impairment resulting from torsion of the contralateral ovary, the patient declined complete oophorectomy. Instead, partial resection of the left ovary was performed. The surgical findings and in vitro maturation workflow are shown in Figure 4. Preoperatively, the patient received 150 IU of follitropin alfa (Merck Biopharma, Darmstadt, Germany) 68 and 44 h before surgery. No hCG trigger was used in this study. The first resected portion (12.1 g, yellow‐dotted area in Figure 4A) yielded no oocytes, and histological examination later confirmed the absence of follicles. A second resection (4.6 g, red‐dotted area) yielded seven GV‐stage oocytes, four of which retained partial cumulus cell attachment (CC+ group) and three were nearly denuded (CC− group).

Because the number of oocytes retrieved during surgery was far lower than anticipated, the treatment plan, within the framework of the previously obtained consent, was reconsidered intraoperatively with the patient's husband. He elected to allocate all available oocytes to dbcAMP‐enhanced biphasic IVM. Importantly, dbcAMP was used exclusively for in vitro exposure, and no systemic administration was performed. DbcAMP (1 mM; Selleck Chemicals, Houston, TX, USA) was added to the pre‐IVM medium to maintain meiotic arrest in the oocytes. A biphasic culture system was used in this study. After 24 h of pre‐IVM and 24 h of IVM, two MII oocytes were obtained from the CC+ group (maturation rate: 50%) and one metaphase I (MI) oocyte from the CC− group (maturation rate: 33%). ICSI of MII oocytes was performed using frozen sperm, which yielded two pronuclei (2PN) zygotes in the CC+ group. The fertilized MII oocytes measured 106 × 106 and 106 × 109 μm, which were smaller than the average MII diameter (116.2 μm) of PCOS patients in our clinic (see Table SS1). The remaining oocyte (CC−) resulted in a 0PN zygote.

An 8‐cell Veeck Grade 2 embryo was cryopreserved on day 3. The other embryo that exhibited direct cleavage was cultured to the blastocyst stage, arrested at the 7‐cell stage, and then discarded. Two months later, frozen embryo transfer (FET) was performed during a hormone‐replacement cycle following Holmström therapy. At 4 weeks and 1 day of gestation, the serum hCG level was 157 mIU/mL. Ultrasonography confirmed an intrauterine pregnancy. Subsequently, prenatal care was transferred to a local hospital. At 39 weeks and 5 days of gestation, the patient underwent emergency cesarean section because of labor arrest caused by cephalopelvic disproportion and fetal distress. A healthy male infant weighing 3,228 g was delivered with an Apgar score of 8/9 and an umbilical artery pH of 7.293. No congenital anomalies were observed. The remaining ovary exhibited a similar lobulated and hypertrophic appearance, but no malignant features.

## 3 Discussion

To the best of our knowledge, this report describes the first documented live birth resulting from dbcAMP‐enhanced biphasic IVM of ovarian tissue‐derived oocytes, achieved through OTO in a patient with OF. Notably, a biochemical pregnancy was achieved in the same patient previously only using conventional hCG‐primed OTO‐IVM, highlighting the potential benefits of dbcAMP‐enhanced biphasic IVM when OTO‐obtained oocytes have limited granulosa cell support, such as in fibrotic or mechanically retrieved ovarian tissues.

### 3.1 Strategies for Infertility Treatment in Ovarian Fibromatosis

The average age of OF onset is approximately 25 years, and it is often discovered incidentally during evaluation for abdominal distension or ovarian torsion. Bilateral involvement is common [1, 5]. Diagnosis is generally established by the characteristic “black garland sign” on MRI and confirmatory histopathology [4]. Our findings from this case were consistent with previous reports in terms of age at diagnosis and imaging findings, but demonstrated unusually extensive bilateral ovarian enlargement, filling the pelvic cavity [10]. Although OF occurs during the reproductive period, its impact on fertility remains poorly documented. Based on previous reports [2, 3, 4, 11, 12, 13], menstrual irregularities are observed in approximately half of the cases, with some showing hyperandrogenic features [12]. In our patient, the detection of multiple small follicles, elevated LH levels, menstrual irregularities, and high AMH levels met the diagnostic criteria for PCOS, suggesting a pathological overlap rather than coincidence. Careful hormonal evaluation is warranted in patients with OFs who present with menstrual abnormalities. Conventional ovulation induction, ovarian drilling, and transvaginal oocyte retrieval are often technically unfeasible when the ovaries are markedly enlarged and firm. Under these circumstances, OTO‐IVM may be the only practical fertility‐preserving strategy.

### 3.2 Limitations of hCG‐Primed OTO‐IVM in Ovarian Fibromatosis

OTO‐IVM has been used for fertility preservation, often alongside ovarian tissue cryopreservation, or for controlled oocyte retrieval from ovarian tumors [14]. However, reports of live births remain rare because of the limited oocyte yield and low IVM maturation rates [6, 15, 16, 17], underscoring the need for improved protocols. We have previously achieved live births using hCG‐primed OTO‐IVM in a patient with a borderline ovarian tumor [17] and applied a similar approach. However, in the present OF case, the oocyte retrieval process itself presented significant difficulties due to the extensive fibrosis and firmness of the ovarian tissue.

In OTO‐IVM, oocytes are typically retrieved using an 18‐G needle under visual or ultrasound guidance; however, this is challenging in unstimulated ovaries, where follicles measure only 5–9 mm. OF appears as lobulated ovarian tumors [5, 12], and fibrosis increases ovarian rigidity, making transvaginal aspiration impractical because the follicles are not visible on the surface [3, 4, 12]. In cases of concurrent ovarian cryopreservation, oocytes are sometimes found in the culture medium after cortical incisions [18]. Therefore, we employed a tissue dicing and agitation technique to isolate oocytes from the resected ovarian tissue. Despite resecting approximately 5% of the ovary, 89 oocytes were retrieved, supporting the effectiveness of this method over conventional follicular aspiration for OF.

Although several oocytes were retrieved during the first OTO‐IVM cycle, difficulties were encountered during IVM, fertilization, and cleavage. This may be due to the variable follicle sizes during surgery and the collection of oocytes from smaller follicles through tissue slicing. Even when IVM is planned for PCOS, oocyte maturity at retrieval depends on the follicle size: 6.9% for ≤ 10 mm, 10.6% for 10–14 mm, and 15.1% for ≥ 14 mm [19]. In this case, follicles approximately 10 mm in size may have developed despite the lack of stimulation. However, in the first OTO‐IVM cycle, the maturation rate of immature oocytes remained low (20.6%), which was markedly lower than the reported 35%–65% maturation rate in conventional OTO‐IVM [18], possibly because the oocytes were collected from small follicles after the ovarian tissue was diced. Furthermore, the incidence of direct cleavage among mature oocytes was high (84.6%) compared to the 9.4% reported in conventional transvaginal oocyte retrieval [20], leading to poor embryo quality. We considered this to be a negative effect of the hCG trigger, as the retrieved oocytes were likely derived from smaller follicles that were less responsive to hCG.

hCG priming before transvaginal oocyte retrieval aims to improve oocyte maturation rates [21]. However, despite the promotion of nuclear maturation, studies on patients with PCOS have reported no significant improvements in pregnancy or live birth rates [22]. This may be due to insufficient LH receptor expression in follicles < 5 mm [23]. In addition, administration of a high dose of hCG to a cohort with small antral follicles has been reported to induce partial or inappropriate maturation [24, 25]. These data show that the major problems in hCG‐primed IVM involve small follicles with minimal granulosa cell attachment to the retrieved oocytes. This finding emphasizes the need for a biphasic IVM approach, specifically the CAPA‐IVM approach.

### 3.3 Efficacy of CAPA‐IVM

A major limitation of conventional IVM is the asynchronous nuclear and cytoplasmic maturation, which reduces the developmental competence of oocytes. To address this issue, biphasic IVM strategies have been developed to delay nuclear maturation, accelerate cytoplasmic maturation, and improve synchronization [7, 26, 27]. Sanchez et al. developed a CNP‐mediated pre‐IVM in which CNP maintains GV‐stage meiotic arrest, enabling cytoplasmic maturation before nuclear maturation [7]. This biphasic culture system, termed “CAPA‐IVM,” has been clinically applied, and improvements in oocyte maturation rates (49.0% to 63.6%), blastocyst formation, and clinical pregnancy rates have been reported [27]. However, the effectiveness of CAPA‐IVM relies on the presence of functional granulosa cells surrounding the oocytes, which is a major limitation in cases such as ours, where granulosa cell attachment is minimal.

### 3.4 Advantages of dbcAMP Over CNP in CAPA‐IVM

Denuded oocytes or those with minimal granulosa cell support generally exhibit poor developmental potential in conventional IVM systems [28]. CNP‐based pre‐IVM relies on intact gap junction communication between granulosa cells and oocytes to sustain intra‐oocyte cAMP levels; therefore, its efficacy is largely limited to oocytes derived from medium‐ to large‐sized follicles. While CNP‐based CAPA‐IVM has improved maturation and developmental outcomes in oocytes from small antral follicles with intact cumulus investment, it does not address the problem of denuded or minimally cumulus‐enclosed oocytes, which therefore remain of low maturation and fertilization potential and are typically excluded from ART procedures [24].

dbcAMP represents a conceptually distinct strategy. As a membrane‐permeable cAMP analog, dbcAMP acts directly within the oocyte, independently of cumulus‐oocyte signaling, to maintain meiotic arrest and facilitate cytoplasmic maturation [29]. Evidence from cattle, sheep, and pigs suggests that dbcAMP‐based pre‐IVM can improve mitochondrial function, reduce oxidative stress, increase intracellular glutathione, and support more synchronized nuclear‐cytoplasmic maturation, mechanisms that are associated with enhanced developmental competence [9, 29, 30, 31]. These mechanistic advantages have contributed to the widespread adoption of dbcAMP‐mediated biphasic IVM in livestock ART, where it has consistently improved blastocyst formation and embryo viability.

In the first OTO‐IVM cycle, immature oocytes showed a low maturation rate, and most embryos exhibited direct cleavage, suggesting that nuclear‐cytoplasmic asynchrony, likely related to premature nuclear maturation after hCG exposure, compromised developmental competence. In contrast, although the number of cleaved oocytes was limited, dbcAMP‐enhanced biphasic IVM may have better maintained meiotic arrest and supported cytoplasmic maturation, even in oocytes with minimal granulosa cell support. Although causality cannot be established from a single case, these biological mechanisms provide a plausible explanation for why only the second cycle yielded a normally cleaving embryo that resulted in the live birth described in this report.

### 3.5 Safety Considerations

Although dbcAMP‐based pre‐IVM has demonstrated promising biological effects in several mammalian species, its safety profile in humans remains unclear. In pigs, short‐term exposure to dbcAMP during pre‐IVM has not been associated with impaired fertilization, abnormal embryogenesis, or reduced blastocyst development [32, 33]. Notably, porcine models using concentrations comparable to those in our protocol have reported normal gestational outcomes, including the birth of healthy piglets without external malformations, abnormal birth weights, or altered gestational length [34]. However, even in animal models, long‐term offspring data remain scarce, and no species has undergone systematic multigenerational follow‐up. Therefore, extrapolation of dbcAMP safety from animals to humans should be approached with caution.

## 4 Conclusion

To the best of our knowledge, this is the first reported live birth following dbcAMP‐enhanced biphasic IVM of ovarian tissue‐derived oocytes. Compared with the previous hCG‐primed OTO‐IVM cycle, the dbcAMP‐based approach appeared to mitigate the challenges associated with limited granulosa cell support, suggesting a potentially granulosa cell‐independent option for patients with atypical ovarian pathology or polycystic ovary‐related infertility.

## Funding

No funding was received for this work.

## Ethics Statement

Human Rights Statements and Informed Consent: All procedures followed were in accordance with the ethical standards of the responsible committee on human experimentation (Institutional Review Board of Shinshu University Hospital, Approval No. B0981; June 10, 2024) and the Helsinki Declaration of 1964 and its later amendments. This study, including the initial hCG‐primed ovarian tissue oocyte in vitro maturation (OTO‐IVM) cycle, was reviewed and approved by the Institutional Review Board of Shinshu University Hospital (Approval No. B0644; July 12, 2021). The subsequent OTO‐IVM cycle, performed using dibutyryl‐cAMP‐enhanced biphasic in vitro maturation of ovarian tissue‐derived oocytes, was specifically reviewed and approved by the same Institutional Review Board (Approval No. B0981; June 10, 2024).

## Consent

Written informed consent for participation and publication of clinical details and images was obtained from the patient.

## Conflicts of Interest

The authors declare no conflicts of interest.

## Supporting information


**Data S1:** Supporting Information.

**Table S1:** Outcomes of Retrieved Oocytes After dbcAMP‐enhanced CAPA‐IVM. This table summarizes the sizes and outcomes of oocytes retrieved from resected ovarian tissues after dbcAMP‐enhanced CAPA‐IVM. On average, the mature oocytes were smaller than those typically observed. Mature oocytes from the cumulus cell‐attached group were successfully fertilized, leading to the cryopreservation of one cleavage‐stage embryo.

**Figure S1:** Regulation of intra‐oocyte cAMP levels to maintain meiotic arrest.
Schematic diagram illustrating two key mechanisms that sustain high intra‐oocyte cAMP levels during the pre‐IVM phase.

**Figure S2:** Clinical timeline of the patient's treatment and outcome.
The timeline illustrates the patient's clinical course, highlighting the key surgical procedures, fertility treatments, and outcomes. At age 24, laparoscopy‐assisted ovarian tumor resection was performed for histological confirmation of ovarian fibromatosis (OF). Subsequent fertility treatments included two ovarian tissue oocyte in vitro maturation (OTO‐IVM) cycles: an hCG‐primed IVM cycle at age 25, which resulted in biochemical pregnancy, and a dbcAMP‐enhanced CAPA‐IVM cycle at age 28, which led to the transfer of a 5‐cell Veeck Grade 3 embryo and the delivery of a healthy male infant at 39 weeks and 5 days via cesarean section.

## Data Availability

The data that support the findings of this study are available on request from the corresponding author. The data are not publicly available due to privacy or ethical restrictions.

## References

[1] R. H. Young and R. E. Scully , “Fibromatosis and Massive Edema of the Ovary, Possibly Related Entities: A Report of 14 Cases of Fibromatosis and 11 Cases of Massive Edema,” International Journal of Gynecological Pathology 3 (1984): 153–178, 10.1097/00004347-198402000-00005. 6490313

[2] P. F. Montoriol and B. Bayol , “Ovarian Fibromatosis: The Black Garland Sign,” Diagnostic and Interventional Imaging 101 (2020): 259–260, 10.1016/j.diii.2019.10.004. 31711935

[3] M. Bazot , C. Salem , A. Cortez , J. M. Antoine , and E. Daraï , “Imaging of Ovarian Fibromatosis,” American Journal of Roentgenology 180 (2003): 1288–1290, 10.2214/ajr.180.5.1801288. 12704039

[4] M. Santos Urios , C. García Espasa , and L. Concepción Aramendía , “Ovarian Fibromatosis: The Black Garland Sign,” Radiología ‐ English Edition 64 (2022): 164–168, 10.1016/j.rxeng.2020.11.009. 35504682

[5] A. Takeda , K. Watanabe , and W. Koike , “Ovarian Fibromatosis Associated With Large Pedunculated Fibroma in a 30‐Year‐Old Woman: A Rare Coincidence or Variant?,” Journal of Obstetrics and Gynaecology Research 50 (2024): 270–274, 10.1111/jog.15830. 37968569

[6] I. Segers , E. Bardhi , I. Mateizel , et al., “Live Births Following Fertility Preservation Using In‐Vitro Maturation of Ovarian Tissue Oocytes,” Human Reproduction 35 (2020): 2026–2036, 10.1093/humrep/deaa175. 32829388

[7] F. Sánchez , F. Lolicato , S. Romero , et al., “An Improved IVM Method for Cumulus‐Oocyte Complexes From Small Follicles in Polycystic Ovary Syndrome Patients Enhances Oocyte Competence and Embryo Yield,” Human Reproduction 32 (2017): 2056–2068, 10.1093/humrep/dex262. 28938744

[8] S. Sugimura , T. Yamanouchi , M. G. Palmerini , Y. Hashiyada , K. Imai , and R. B. Gilchrist , “Effect of Pre‐In Vitro Maturation With cAMP Modulators on the Acquisition of Oocyte Developmental Competence in Cattle,” Journal of Reproduction and Development 64 (2018): 233–241, 10.1262/jrd.2018-009. 29503399 PMC6021610

[9] G. Ramos Leal , C. A. Santos Monteiro , J. M. G. Souza‐Fabjan , et al., “Role of cAMP Modulator Supplementations During Oocyte in Vitro Maturation in Domestic Animals,” Animal Reproduction Science 199 (2018): 1–14, 10.1016/j.anireprosci.2018.11.002. 30449707

[10] S. C. Hong , M. Y. Kim , J. M. Kim , and S. O. Hwang , “Bilateral Ovarian Fibromatosis in a Postmenopausal Female: A Case Report With Emphasis on MRI Findings and Differential Diagnosis,” Journal of the Korean Society of Radiology 85 (2024): 970–975, 10.3348/jksr.2024.0010. 39416319 PMC11473983

[11] E. L. Spurrell , Y. C. Yeo , T. P. Rollason , and I. R. Judson , “A Case of Ovarian Fibromatosis and Massive Ovarian Oedema Associated With Intra‐Abdominal Fibromatosis, Sclerosing Peritonitis and Meig's Syndrome,” Sarcoma 8 (2004): 113–121, 10.1080/13577140400011136. 18521405 PMC2395617

[12] L. S. Onderoglu , M. Gültekin , P. Dursun , et al., “Bilateral Ovarian Fibromatosis Presenting With Ascites and Hirsutism,” Gynecologic Oncology 94 (2004): 223–225, 10.1016/j.ygyno.2004.03.042. 15262147

[13] N. Bakshi and V. Kaushal , “Ovarian fibromatosis,” Journal of Obstetrics and Gynaecology of India 64 (2014): 368–369, 10.1007/s13224-012-0260-7. 25368465 PMC4199422

[14] I. Segers , I. Mateizel , K. Wouters , et al., “Ovarian Tissue Oocyte‐In Vitro Maturation for Fertility Preservation,” Journal of Visualized Experiments 207 (2024): e65255, 10.3791/65255. 38829044

[15] E. B. Prasath , M. L. H. Chan , W. H. W. Wong , et al., “First Pregnancy and Live Birth Resulting From Cryopreserved Embryos Obtained From in Vitro Matured Oocytes After Oophorectomy in an Ovarian Cancer Patient,” Human Reproduction 29 (2014): 276–278, 10.1093/humrep/det420. 24327539

[16] P. S. Uzelac , A. A. Delaney , G. L. Christensen , H. C. L. Bohler , and S. T. Nakajima , “Live Birth Following in Vitro Maturation of Oocytes Retrieved From Extracorporeal Ovarian Tissue Aspiration and Embryo Cryopreservation for 5 Years,” Fertility and Sterility 104 (2015): 1258–1260, 10.1016/j.fertnstert.2015.07.1148. 26297647

[17] S. Higuchi , T. Miyamoto , K. Oka , H. Kobara , and T. Shiozawa , “Successful Pregnancy Using Immature Oocytes Retrieved From Resected Borderline Ovarian Tumor: A Case Report and Literature Review,” Contraception and Reproductive Medicine 9 (2024): 24, 10.1186/s40834-024-00285-9. 38755650 PMC11097572

[18] C. De Roo and K. Tilleman , “In Vitro Maturation of Oocytes Retrieved From Ovarian Tissue: Outcomes From Current Approaches and Future Perspectives,” Journal of Clinical Medicine 10 (2021): 4680, 10.3390/jcm10204680. 34682803 PMC8540978

[19] W. Y. Son and S. L. Tan , “Laboratory and Embryological Aspects of hCG‐Primed in Vitro Maturation Cycles for Patients With Polycystic Ovaries,” Human Reproduction Update 16 (2010): 675–689, 10.1093/humupd/dmq014. 20504873

[20] M. Tam Le , T. Van Nguyen , T. Thanh Nguyen , et al., “Does Polycystic Ovary Syndrome Affect Morphokinetics or Abnormalities in Early Embryonic Development?,” European Journal of Obstetrics & Gynecology and Reproductive Biology: X 3 (2019): 100045, 10.1016/j.eurox.2019.100045. 31403129 PMC6687388

[21] X. Zheng , L. Wang , X. Zhen , Y. Lian , P. Liu , and J. Qiao , “Effect of hCG Priming on Embryonic Development of Immature Oocytes Collected From Unstimulated Women With Polycystic Ovarian Syndrome,” Reproductive Biology and Endocrinology 10 (2012): 40, 10.1186/1477-7827-10-40. 22621829 PMC3499152

[22] Y. Lin , X. Zheng , C. Ma , et al., “Human Chorionic Gonadotropin Priming Does Not Improve Pregnancy Outcomes of PCOS‐IVM Cycles,” Front Endocrinol ‐ Lausanne 11 (2020): 279, 10.3389/fendo.2020.00279. 32425891 PMC7204525

[23] J. V. Jeppesen , S. G. Kristensen , M. E. Nielsen , et al., “LH‐Receptor Gene Expression in Human Granulosa and Cumulus Cells From Antral and Preovulatory Follicles,” Journal of Clinical Endocrinology and Metabolism 97 (2012): E1524–E1531, 10.1210/jc.2012-1427. 22659248 PMC3410279

[24] J. Smitz , F. Sánchez , S. Romero , et al., “Human Oocyte Capacitation Culture: Essential Step Toward Hormone‐Free Assisted Reproductive Technology,” Reproductive Medicine and Biology 24 (2025): e12640, 10.1002/rmb2.12640. 40078334 PMC11897612

[25] R. B. Gilchrist , T. M. Ho , M. De Vos , et al., “A Fresh Start for IVM: Capacitating the Oocyte for Development Using Pre‐IVM,” Human Reproduction Update 30, no. 1 (2024): 3–25, 10.1093/humupd/dmad023. 37639630

[26] F. Sanchez , A. H. Le , V. N. A. Ho , et al., “Biphasic in Vitro Maturation (CAPA‐IVM) Specifically Improves the Developmental Capacity of Oocytes From Small Antral Follicles,” Journal of Assisted Reproduction and Genetics 36 (2019): 2135–2144, 10.1007/s10815-019-01551-5. 31399916 PMC6823411

[27] L. N. Vuong , A. H. Le , V. N. A. Ho , et al., “Live Births After Oocyte in Vitro Maturation With a Prematuration Step in Women With Polycystic Ovary Syndrome,” Journal of Assisted Reproduction and Genetics 37 (2020): 347–357, 10.1007/s10815-019-01677-6. 31902102 PMC7056678

[28] J. Zhang , Q. Wei , J. Cai , X. Zhao , and B. Ma , “Effect of C‐Type Natriuretic Peptide on Maturation and Developmental Competence of Goat Oocytes Matured in Vitro,” PLoS One 10 (2015): e0132318, 10.1371/journal.pone.0132318. 26151446 PMC4511268

[29] H. R. Namkung , S. B. Jung , S. Y. Nam , et al., “Temporal Optimization of Meiotic Arrest for Enhancing Oocyte Maturity During in Vitro Maturation of Porcine Median Antral Follicles,” Reproductive Biology 25 (2025): 100987, 10.1016/j.repbio.2024.100987. 39644800

[30] M. Navarro , T. Fanti , N. M. Ortega , et al., “The Simulated Physiological Oocyte Maturation (SPOM) System Enhances Cytoplasmic Maturation and Oocyte Competence in Cattle,” Animals ‐ Basel 14 (2024): 1893, 10.3390/ani14131893. 38998004 PMC11240716

[31] H. J. Li , M. L. Sutton‐McDowall , X. Wang , S. Sugimura , J. G. Thompson , and R. B. Gilchrist , “Extending Prematuration With cAMP Modulators Enhances the Cumulus Contribution to Oocyte Antioxidant Defence and Oocyte Quality via Gap Junctions,” Human Reproduction 31 (2016): 810–821, 10.1093/humrep/dew020. 26908844

[32] M. A. Bagg , M. B. Nottle , C. G. Grupen , and D. T. Armstrong , “Effect of Dibutyryl cAMP on the cAMP Content, Meiotic Progression, and Developmental Potential of in Vitro Matured Pre‐Pubertal and Adult Pig Oocytes,” Molecular Reproduction and Development 73 (2006): 1326–1332, 10.1002/mrd.20555. 16865720

[33] T. Somfai and Y. Hirao , “Synchronization of in Vitro Maturation in Porcine Oocytes,” in Methods in Molecular Biology, vol. 1817. Humana Press. 10.1007/978-1-4939-6603-5_16. 27815908

[34] Y. Akaki , K. Yoshioka , M. Noguchi , et al., “Successful Piglet Production in a Chemically Defined System for In‐Vitro Production of Porcine Embryos: Dibutyryl Cyclic AMP and Epidermal Growth Factor‐Family Peptides Support In‐Vitro Maturation of Oocytes in the Absence of Gonadotropins,” Journal of Reproduction and Development 52 (2006): 447–456, 10.1262/jrd.17088. 19444007

