# Supplementary material for: Live Birth Following Dibutyryl‐cAMP‐Enhanced Biphasic in Vitro Maturation of Ovarian Tissue Oocytes From a Patient With Ovarian Fibromatosis: A First Report
Source: Reprod Med Biol. 2026 Mar 11;25(1):e70024. doi: 10.1002/rmb2.70024 (PMC12977298; doi:10.1002/rmb2.70024)
Supplement: Supplementary file 1 — Data S1: Supporting Information. [file RMB2-25-e70024-s003.docx]

***Supplementary Material***

Live Birth Following Dibutyryl-cAMP-enhanced Biphasic In Vitro Maturation of Ovarian Tissue Oocytes from a Patient with Ovarian Fibromatosis: A First Report

**Running title**

Live Birth After dbcAMP-enhanced Biphasic IVM of Ovarian Tissue Oocytes

Shotaro Higuchi*, M.D., Ph.D. ^a^, Tsutomu Miyamoto, M.D., Ph.D. ^a^, Miho Mochizuki^b^, Tamae Fukushima^b^, Koichi Ida, M.D., Ph.D. ^a^, Hisanori Kobara, M.D., Ph.D. ^a^, Ayumi Ohya, M.D., Ph.D. ^c^, Yasunari Fujinaga, M.D., Ph.D. ^c^, and Tanri Shiozawa, M.D., Ph.D. ^a^

^a^ Department of Obstetrics and Gynecology, Shinshu University School of Medicine, Matsumoto, Nagano, Japan.

^b^ Center for Reproductive Medicine, Shinshu University Hospital, Matsumoto, Nagano, Japan.

^c^ Department of Radiology, Shinshu University School of Medicine, Matsumoto, Nagano, Japan.

***Corresponding Author**:

Shotaro Higuchi, M.D., Ph.D.
Department of Obstetrics and Gynecology, Shinshu University School of Medicine
3-1-1 Asahi, Matsumoto, Nagano 390-8621, Japan
Phone: +81-263-37-2719
Email: [**taro_hig@shinshu-u.ac.jp**](mailto:taro_hig@shinshu-u.ac.jp)

ORCID: https://orcid.org/0009-0001-9795-9124

**Supplementary material**

**Supplemental Fig. 1. Regulation of intra-oocyte cAMP levels to maintain meiotic arrest**

Schematic diagram illustrating two key mechanisms that sustain high intra-oocyte cAMP levels during the pre-IVM phase.

1) FSH (follicle-stimulating hormone) promotes cAMP production in granulosa cells, which diffuses through gap junctions into cumulus cells and oocytes.

2) C-type natriuretic peptide (CNP), secreted by granulosa cells or administered exogenously, binds to NPR2 (natriuretic peptide receptor 2) in cumulus cells. This activates guanylyl cyclase to produce cGMP (cyclic guanosine monophosphate, which enters the oocytes via gap junctions and inhibits PDE3A (phosphodiesterase 3A), thereby preventing cAMP degradation.

Elevated cAMP levels suppress the activation of maturation-promoting factors (MPF), maintain oocytes in the germinal vesicle (GV) stage. CNPs are commonly used in human CAPA-IVM; however, their effectiveness may be limited to oocytes with insufficient granulosa or cumulus cell support. Dibutyryl-cAMP directly sustains intraoocyte cAMP levels without relying on granulosa cells.

**Supplemental Fig. 2. Clinical timeline of the patient’s treatment and outcome**

The timeline illustrates the patient’s clinical course, highlighting the key surgical procedures, fertility treatments, and outcomes. At age 24, laparoscopy-assisted ovarian tumor resection was performed for histological confirmation of ovarian fibromatosis (OF). Subsequent fertility treatments included two ovarian tissue oocyte in vitro maturation (OTO-IVM) cycles: an hCG-primed IVM cycle at age 25, which resulted in biochemical pregnancy, and a dbcAMP-enhanced CAPA-IVM cycle at age 28, which led to the transfer of a 5-cell Veeck Grade 3 embryo and the delivery of a healthy male infant at 39 weeks and 5 days via cesarean section.

**Supplemental Table 1** **Outcomes of Retrieved Oocytes After dbcAMP-enhanced CAPA-IVM**

This table summarizes the sizes and outcomes of oocytes retrieved from resected ovarian tissues after dbcAMP-enhanced CAPA-IVM. On average, the mature oocytes were smaller than those typically observed. Mature oocytes from the cumulus cell-attached group were successfully fertilized, leading to the cryopreservation of one cleavage-stage embryo.
