# Supplementary material for: Live Birth Following Dibutyryl‐cAMP‐Enhanced Biphasic in Vitro Maturation of Ovarian Tissue Oocytes From a Patient With Ovarian Fibromatosis: A First Report
Source: Reprod Med Biol. 2026 Mar 11;25(1):e70024. doi: 10.1002/rmb2.70024 (PMC12977298; doi:10.1002/rmb2.70024)
Supplement: Supplementary file 2 — Table S1: Outcomes of Retrieved Oocytes After dbcAMP‐enhanced CAPA‐IVM. This table summarizes the sizes and outcomes of oocytes retrieved from resected ovarian tissues after dbcAMP‐enhanced CAPA‐IVM. On average, the mature oocytes were smaller than those typically observed. Mature oocytes from the cumulus cell‐attached group were successfully fertilized, leading to the cryopreservation of one cleavage‐stage embryo. [file RMB2-25-e70024-s004.docx]

**Supplemental Table 1 Outcomes of Retrieved Oocytes After dbcAMP-enhanced CAPA-IVM**

|  | After IVM | Size (μm) | Result of ICSI | Cryopreservation |
| --- | --- | --- | --- | --- |
|  |  |  |  |  |
| Cumulus-cell Attached Group | MⅡ | 106×108 | 2PN | Day 3, 8-cell Veeck 2 |
|  | MⅡ | 106×106 | 2PN | Not blastocyst |
|  | GV | 97×100 |  |  |
|  | Degenerated | ‐ |  | ‐ |
| Cumulus-cell Denuded Group | MⅡ | 110×118 | 0PN | ‐ |
|  | MⅠ | 109×110 |  | ‐ |
|  | GV | 100×100 |  | ‐ |

This table summarizes the sizes and outcomes of oocytes retrieved from resected ovarian tissues after dbcAMP-enhanced CAPA-IVM. On average, the mature oocytes were smaller than those typically observed. Mature oocytes from the cumulus cell-attached group were successfully fertilized, leading to the cryopreservation of one cleavage-stage embryo.
