# Supplementary figures and images for: Live Birth Following Dibutyryl‐cAMP‐Enhanced Biphasic in Vitro Maturation of Ovarian Tissue Oocytes From a Patient With Ovarian Fibromatosis: A First Report
Source: Reprod Med Biol. 2026 Mar 11;25(1):e70024. doi: 10.1002/rmb2.70024 (PMC12977298; doi:10.1002/rmb2.70024)

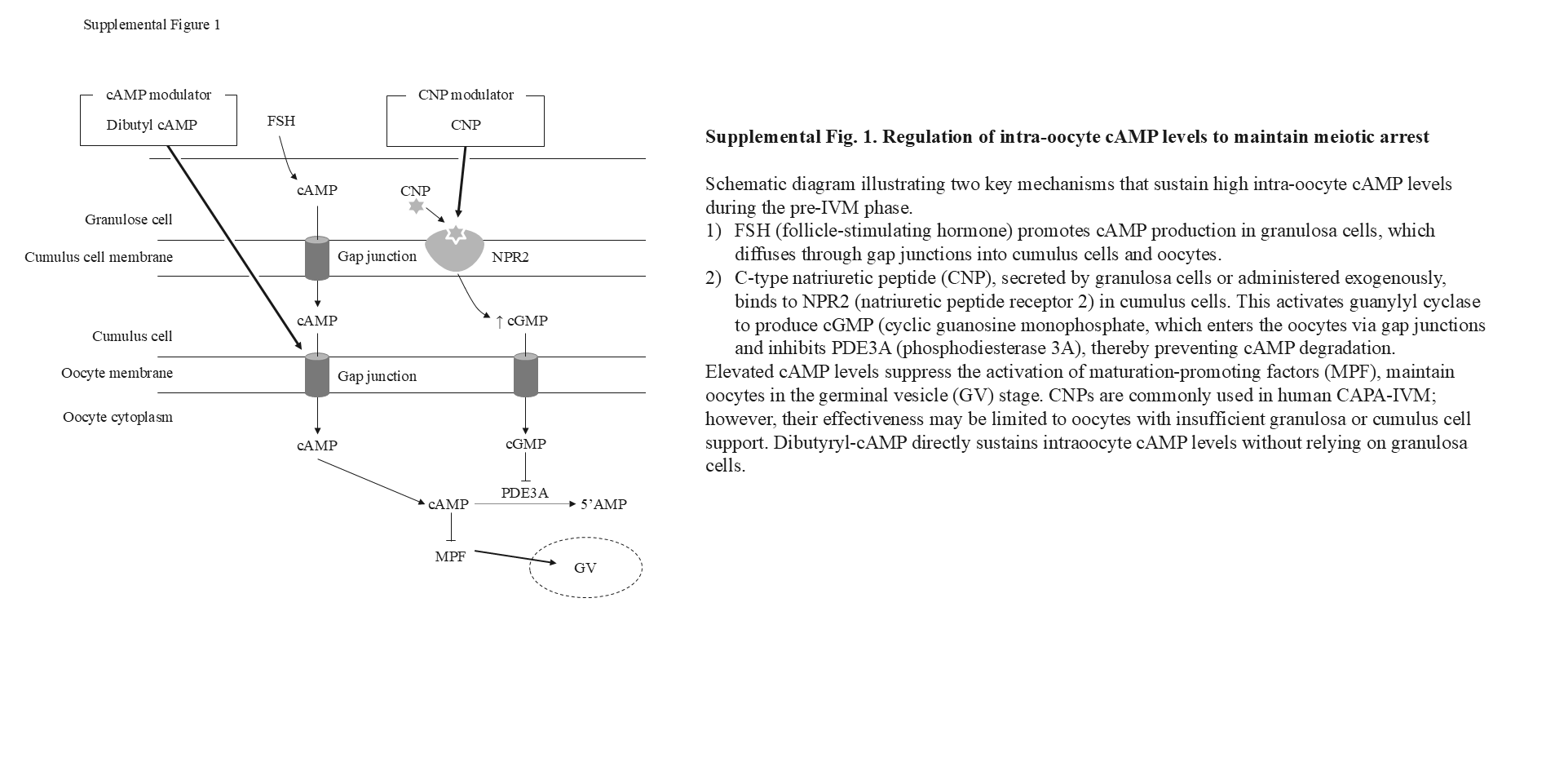

Supplement: Supplementary file 3 — Figure S1: Regulation of intra‐oocyte cAMP levels to maintain meiotic arrest. Schematic diagram illustrating two key mechanisms that sustain high intra‐oocyte cAMP levels during the pre‐IVM phase. [file RMB2-25-e70024-s001.tif]

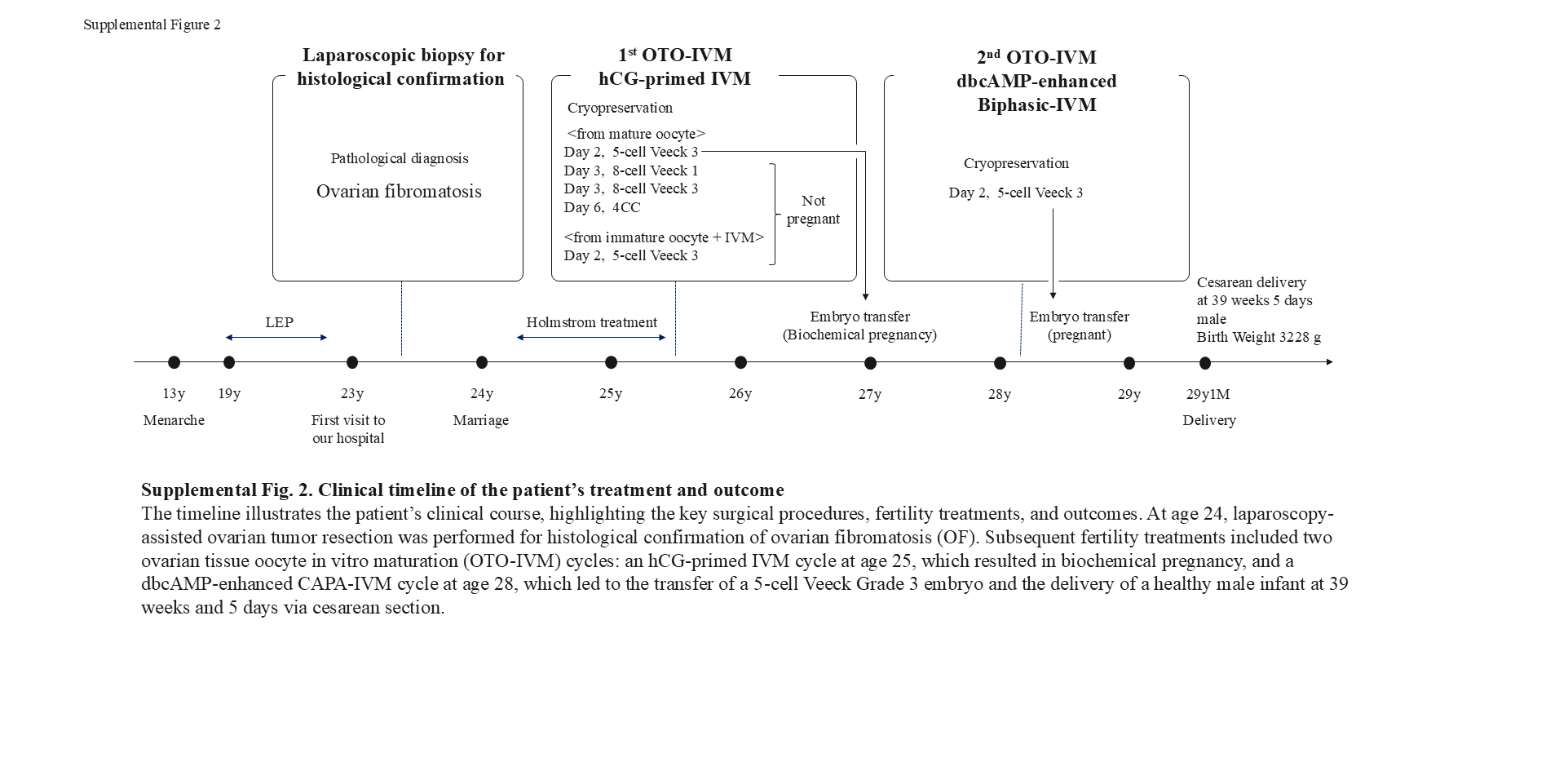

Supplement: Supplementary file 4 — Figure S2: Clinical timeline of the patient's treatment and outcome. The timeline illustrates the patient's clinical course, highlighting the key surgical procedures, fertility treatments, and outcomes. At age 24, laparoscopy‐assisted ovarian tumor resection was performed for histological confirmation of ovarian fibromatosis (OF). Subsequent fertility treatments included two ovarian tissue oocyte in vitro maturation (OTO‐IVM) cycles: an hCG‐primed IVM cycle at age 25, which resulted in biochemical pregnancy, and a dbcAMP‐enhanced CAPA‐IVM cycle at age 28, which led to the transfer of a 5‐cell Veeck Grade 3 embryo and the delivery of a healthy male infant at 39 weeks and 5 days via cesarean section. [file RMB2-25-e70024-s002.tif]
